# Relationships between p14^ARF^ Gene Methylation and Clinicopathological Features of Colorectal Cancer: A Meta-Analysis

**DOI:** 10.1371/journal.pone.0152050

**Published:** 2016-03-21

**Authors:** Zhangjian Zhou, Hao Zhang, Jianguo Lai, Dongmei Diao, Wenhan Li, Chengxue Dang, Yongchun Song

**Affiliations:** Division of Surgical Oncology, The First Affiliated Hospital, Xi'an Jiaotong University, 277 W, Yanta Road, Xi'an, 710061, Shaanxi, China; Thomas Jefferson University, UNITED STATES

## Abstract

We conducted a meta-analysis to explore the relationships between p14^ARF^ gene methylation and clinicopathological features of colorectal cancer (CRC). Databases, including Pubmed, Embase and Cochrane Library, were searched and, finally, a total of 18 eligible researches encompassing 1988 CRC patients were selected. Combined odds ratios (ORs) with 95% confidence intervals (95% CIs) were evaluated under a fixed effects model for absence of heterogeneity. Significant associations were observed between p14^ARF^ gene methylation and tumor location (OR = 2.35, 95% CI: 1.55–3.55, *P* = 0.001), microsatellite instability (MSI) status (OR = 3.28, 95% CI: 2.12–5.07, *P*<0.0001). However, there were no significant associations between p14^ARF^ gene methylation and tumor stage, tumor differentiation. We concluded that p14^ARF^ gene methylation may be significantly associated with tumor location, and MSI status of CRC.

## Introduction

Colorectal cancer (CRC) is still one of the most common cancers worldwide, with about 1.3 million new cases and 700,000 deaths each year [1]. It is well known that genetic and epigenetic alterations both play important roles in the progression of CRC [2, 3]. As one of the most important epigenetic changes, DNA methylation has been noted in the carcinogenesis of many human cancers, including CRC [4–8]. Many previous studies have revealed that aberrant methylation of p14^ARF^ gene is involved in the development of CRC [9–11].

The alterations of P53/MDM2/p14^ARF^ pathway always occur during CRC progression. p14^ARF^, through its effect on MDM2 levels, eventually increases the concentration of p53 protein, another important tumor suppressor that is essential for regulating cell division and apoptosis. By protecting p53 protein, p14^ARF^ helps preventing tumor formation. Therefore, we have reason to think that silencing of p14^ARF^ gene is a possible way to deregulate cell cycle control by interfering the p53 pathway. It has been shown recently that hypermethylation of p14^ARF^ gene is extensively detected in primary CRC, which led to the loss of p14^ARF^ mRNA and protein expression [12–14]. However, the relationships between p14^ARF^ gene methylation and clinicopathological features of CRC remain controversial. Besides, epigenetic markers such as p14^ARF^ gene methylation may be used to more accurately classify subgroups of CRC patients. Therefore, we conducted a meta-analysis to quantify the associations between p14^ARF^ gene methylation and clinicopathological features of CRC.

## Materials and Methods

### Search strategy

Electronic databases, the PubMed, Cochrane Library, and Embase, were manually searched to find those relevant researches published before May 1st, 2015. The terms we used during the search were: “p14”, “p14^ARF^”, “DNA methylation”, “hypermethylation”, “methylation”, “colon”, “rectum”, “colorectal”, “cancer”, and “carcinoma”. In order to get some more potential researches, we also checked the references in each article. All authors’ names and affiliations were attentively screened to avoid repeated data.

### Selection criteria

To identify eligible researches, we used a set of inclusion criteria: (1) Histopathologic information of CRC patients were confirmed by the pathologist review; (2) Methylation analysis of p14^ARF^ gene was performed in primary tumor tissues after operation, not in normal colonic mucosa, serum, and peripheral blood leukocyte of CRC patients; (3) Data with regard to the relationships between p14^ARF^ gene methylation and clinicopathological features of CRC patients was provided, which is conducive to estimating the pooled ORs and 95% CIs; (4) The latest or most comprehensive data was our choice when duplications were published. (5) The splenic flexure was used as the anatomical boundary to define proximal and distal CRC; and (6) Microsatellite instability (MSI) status was assessed by examining five independent genomic sites, including two mononucleotide repeat microsatellites (BAT25 and BAT26) and three dinucleotide repeat microsatellites (D2S123, D5S346 and D17S250) as recommended by the National Cancer Institute Workshop. MSI was positive if two or more of the markers showed instability. When one or none of the markers showed instability, MSI was negative [15]. Two authors (ZJZ and HZ) performed this search work separately and divergence was resolved by discussion with another author (JGL).

### Data extraction

When eligible studies were identified, we extracted necessary data according to these following items: first author’s name, year of publication, geographical location, number of patients, demographic features, clinicopathological features, detection method of methylation, number of p14^ARF^ gene methylated patients and total number of patients in case and control groups.

### Quality assessment

Quality assessment of the selected studies was performed on the basis of the Newcastle-Ottawa Scale (NOS) criteria [16]. The NOS scores were obtained based on three items: selection, comparability, and outcome, and a score of > 6 means high quality.

### Statistical analysis

The meta-analysis and graphics were accomplished using R software version 3.2.0 with the “meta” package. A fixed or random effects model was applied to estimate the combined ORs and 95% CIs. Heterogeneity between included studies was detected using the Cochran’s Q-statistic and *I*^*2*^ test, and a *p* < 0.05 or a result > 50% suggests significant heterogeneity. In this case, the random effects model was chosen; otherwise, the fixed effects model was used [17]. Publication bias was graphically tested by funnel plots and further statistically assessed by Peters test [18]. The trim and fill method was applied to adjust the combined ORs and 95% CIs when publication bias existed. To explore the influence of each single study on the overall estimate, sensitivity analysis was performed by pooling the remaining studies after excluding each single study.

## Results

**Fig 1:**
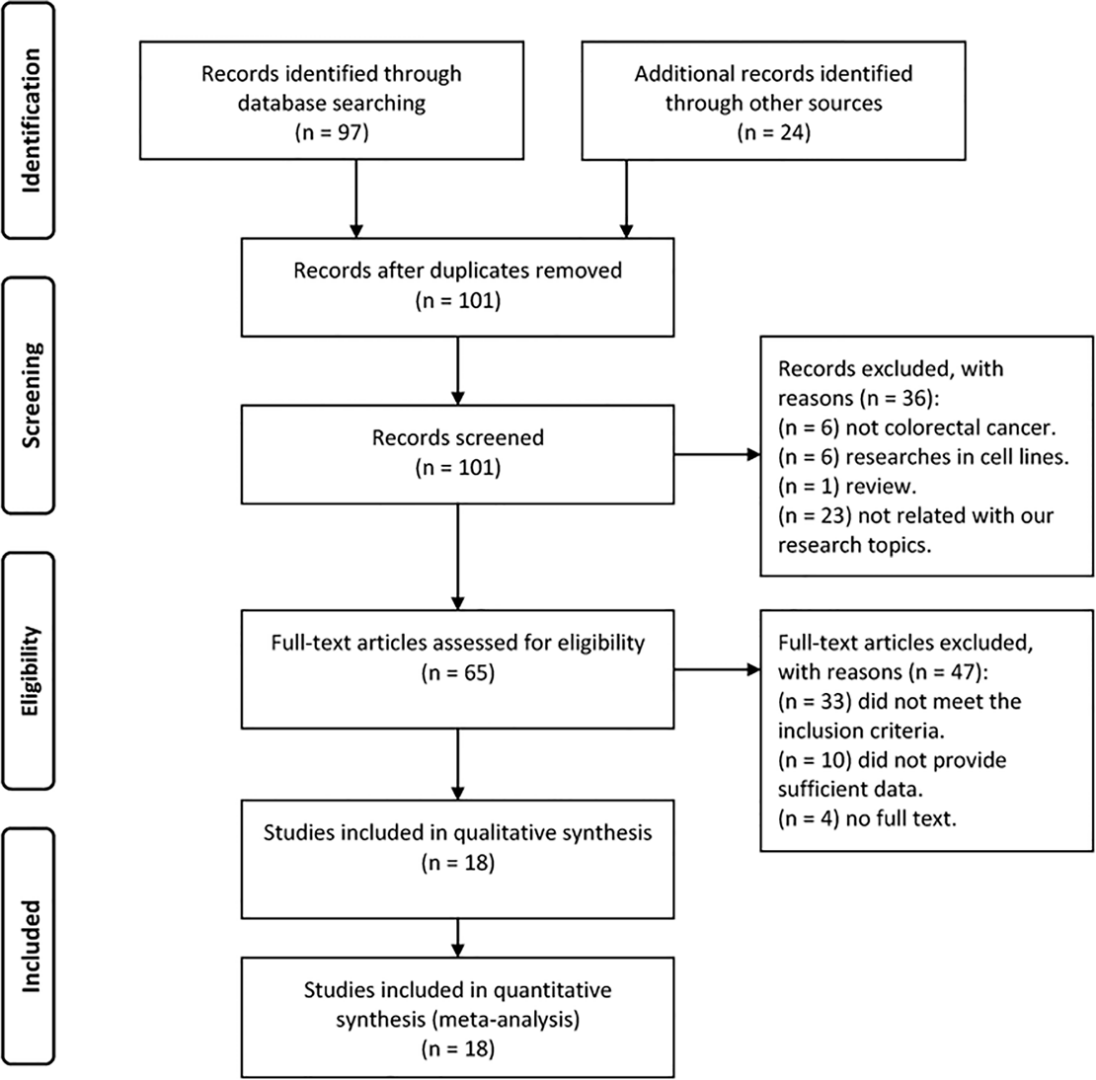
Flow diagram of study selection.

A total of 101 relevant studies were identified for initial review using the described search method. The titles and abstracts of all studies were screened and 36 were initially excluded according to the inclusion criteria. Then, the full texts of the remaining studies were carefully read, and 47 studies were excluded with various reasons. Therefore, 18 studies were finally included in the meta-analyses [9, 11, 14, 19–33]. The process of study selection is shown in Fig 1. The basic characteristics of the 18 included studies are extracted and summarized in Table 1. A total of 1988 CRC patients were involved in this meta-analysis. These 18 studies were published from 2000 to 2014 and were of good quality, with an average NOS score of 6.3 (5–7). Table 2 shows the summary of our meta-analysis results. The fixed effects model was used in this study for absence of significant heterogeneity.

**Table 1:** Basic characteristics of the included studies. MSP, methylation-specific PCR; BGS, bisulfite genomic sequencing; BSSCP, bisulfite single-strand conformation poly-morphism; REP, restriction enzyme-related PCR.

Study	Country	No. of Cases	Gender(M/F)	Age(years)	Method(s)
Chaar I 2014	Tunisia	167	84/83	57	MSP
Nyiraneza C 2012	Belgium	35	16/19	67.4	MSP
Naghibalhossaini F 2011	Iran	110	72/38	—	MSP
Kim JC 2010	Korea	285	168/117	58±10	BGS
Kondo I 2008	Japan	97	57/40	63.9(25–86)	MSP
Derks S 2008	Netherlands	67	34/33	67(44–89)	MSP
Kang MY 2008	Korea	188	115/73	60(21–86)	MSP,BGS
Goel A 2007	America	126	—	—	MSP
Lee M 2006	Korea	65	—	61(27–85)	MSP
Iacopetta B 2006	Australia	199	122/77	—	Methylight
Bai AH 2004	China	47	16/31	72.0±12.7	MSP
Lind GE 2004	Norway	52	25/27	—	MSP
Dominguez G 2003	Spain	95	—	—	MSP
Yamamoto H 2002	Japan	80	—	—	MSP
Hibi K 2002	Japan	86	51/35	60.1	MSP
Burri N 2001	Switzerland	60	34/26	—	REP,MSP,BGS,BSSCP
Zheng S 2000	America	119	60/59	—	MSP
Esteller M 2000	America	110	—	—	MSP

**Table 2:** Summary of pooled results in the meta-analysis. MSI, microsatellite instability.

Groups	No. of studies	OR and 95% CI	*P*	Heterogeneity		Model
				*I*^*2*^ (%)	*P*	
Male vs. Female	10	0.71(0.53–0.95)	0.021	25.5	0.209	Fixed
TNM stage (I & II vs. III & IV)	6	0.98(0.70–1.38)	0.926	0.0	0.833	Fixed
Dukes stage (A & B vs. C & D)	5	1.28(0.80–2.05)	0.299	21.3	0.279	Fixed
Location (Proximal vs. Distal)	5	2.35(1.55–3.55)	0.001	9.7	0.351	Fixed
Well or Moderate vs. Poor or Others	9	0.97(0.61–1.54)	0.900	22.8	0.240	Fixed
MSI positive vs. MSI negative	7	3.28(2.12–5.07)	0.000	43.2	0.103	Fixed
TP53 mutation vs. TP53 wild	5	0.72(0.45–1.14)	0.157	36.8	0.176	Fixed

**Fig 2:**
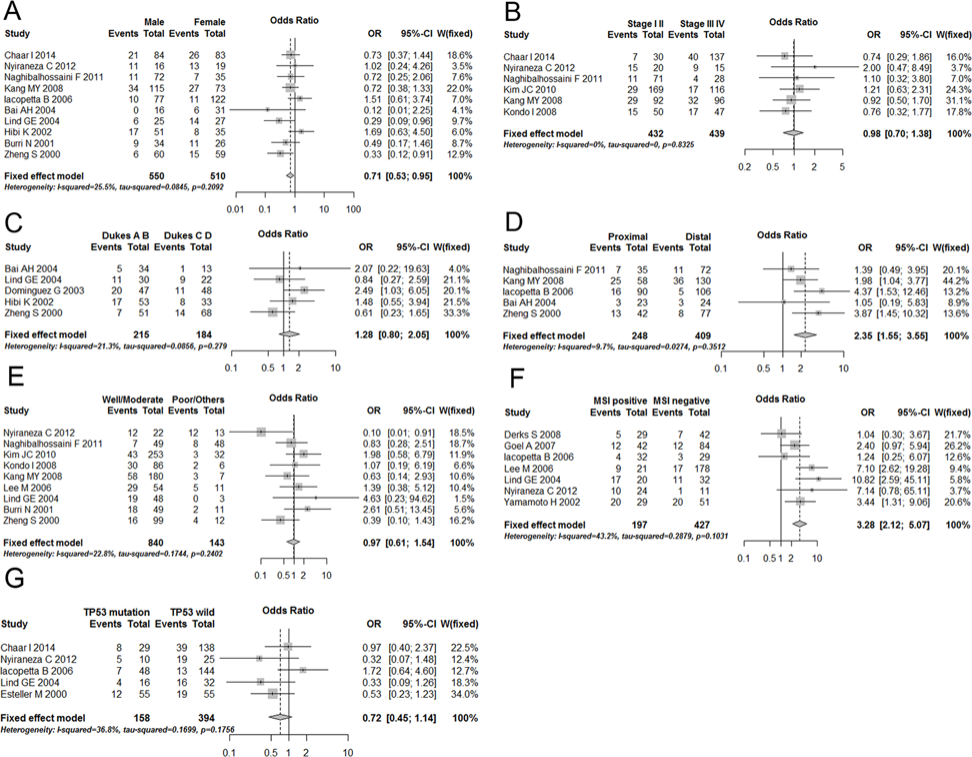
Forest plots for the relationships between p14^ARF^ gene methylation and the clinicopathological features of CRC. (A) gender (male vs. female); (B) TNM stage (I & II vs. III & IV); (C) Dukes stage (A & B vs. C & D); (D) Location (Proximal vs. Distal); (E) tumor differentiation; (F) MSI status; (G) TP53 mutation status. OR, odds ratio; CI, confidence interval; CRC, colorectal cancer

Our results showed that p14^ARF^ gene in samples from female patients was more likely to be methylated than those from male patients (male vs. female: OR = 0.71, 95% CI: 0.53–0.95, *p* = 0.021) (Fig 2A). Age was also considered in this analysis. Due to the different styles of variables used in individual studies, we perform three separate meta-analyses, among which two showed no significant relationship between age and p14^ARF^ methylation in CRC patients while the other one showed the contrary result (S1 Fig). Relationship between p14^ARF^ gene methylation and tumor stage was also evaluated in this study, and neither TNM nor Dukes stage were significantly associated with p14^ARF^ gene methylation (I & II vs. III & IV: OR = 0.98, 95% CI: 0.70–1.38, *p* = 0.926; A & B vs. C & D: OR = 1.28, 95% CI: 0.80–2.05, *p* = 0.299, respectively) (Fig 2B and 2C). As for the CRC tumor location, our results revealed that the classification of proximal vs. distal was significantly correlated with p14^ARF^ gene methylation (OR = 2.35, 95% CI: 1.55–3.55, *p* = 0.001) (Fig 2D). Then, we further explored whether p14^ARF^ gene methylation was related with CRC tumor differentiation, and no significance was found (well or moderate vs. poor or others: OR = 0.97, 95% CI: 0.61–1.54, *p* = 0.900) (Fig 2E). Additionally, MSI positive patients were found to have a higher possibility of p14^ARF^ gene methylation than MSI negative ones (MSI positive vs. MSI negative: OR = 3.28, 95% CI: 2.12–5.07, *p* = 0.0001) (Fig 2F). However, we found no evidence for any significant association between p14^ARF^ gene methylation and TP53 mutation status (TP53 mutation vs. TP53 wild: OR = 0.72, 95% CI: 0.45–1.14, *p* = 0.157) (Fig 2G).

**Fig 3:**
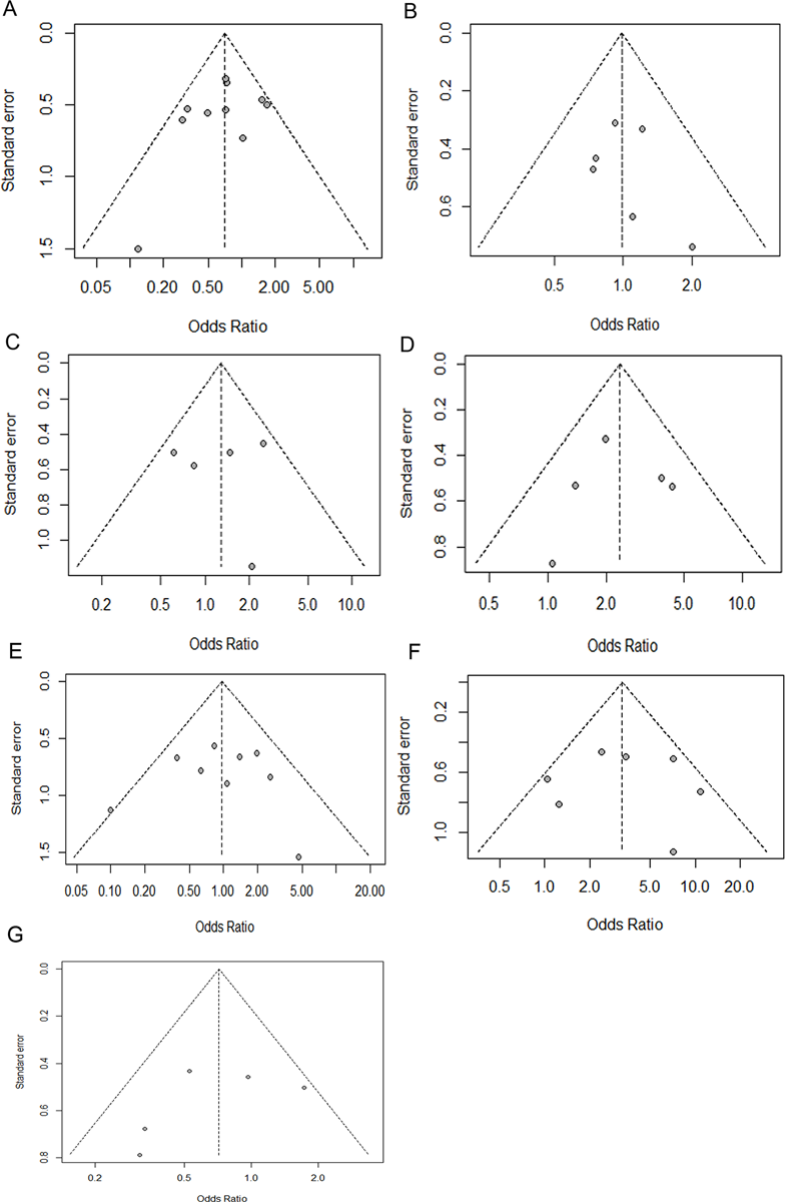
Funnel plots of publication bias. (A) gender (male vs. female); (B) TNM stage (I & II vs. III & IV); (C) Dukes stage (A & B vs. C & D); (D) Location (Proximal vs. Distal); (E) tumor differentiation; (F) MSI status; (G) TP53 mutation status. MSI, microsatellite instability

**Fig 4:**
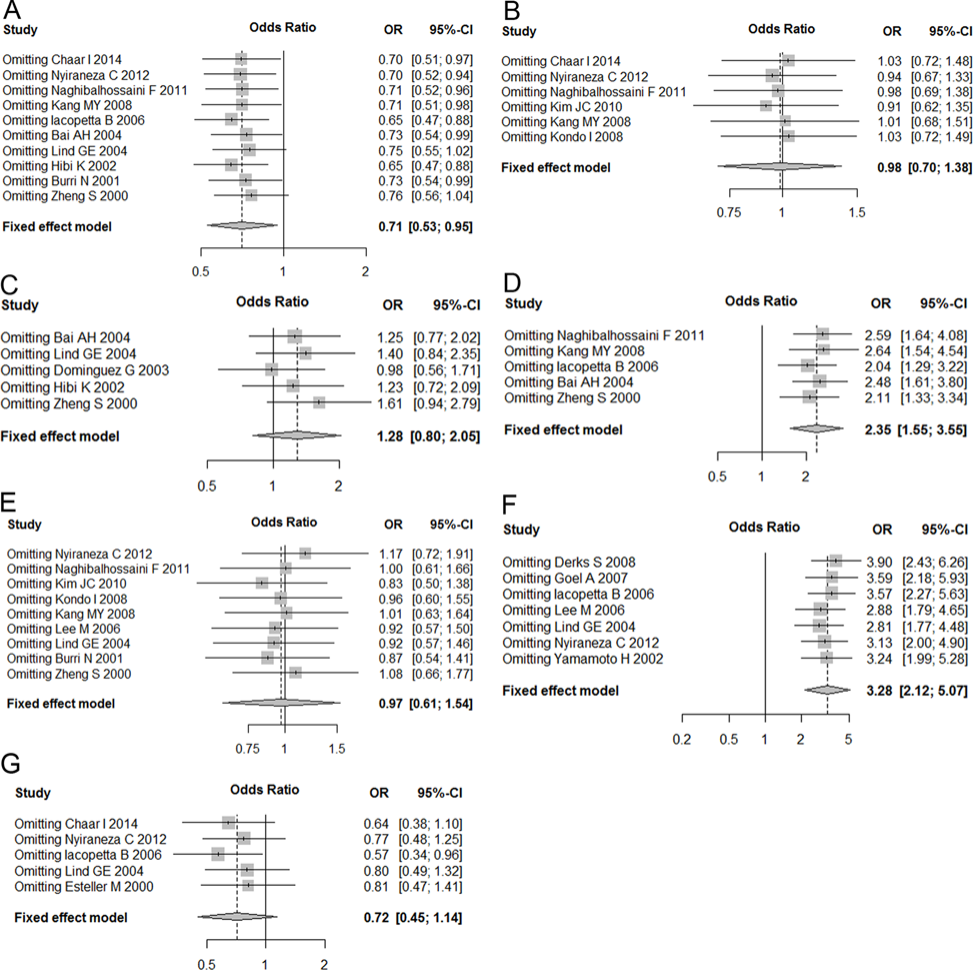
Sensitivity analysis of the influence of omitting each single study on the combined ORs. (A) gender (male vs. female); (B) TNM stage (I & II vs. III & IV); (C) Dukes stage (A & B vs. C & D); (D) Location (Proximal vs. Distal); (E) tumor differentiation; (F) MSI status; (G) TP53 mutation status. MSI, microsatellite instability

Funnel plots were used to explore the publication bias and, as showed in Fig 3, all plots were symmetrical, indicating no significant biases. Peters test further confirmed the symmetry of funnel plots for eligible studies concerning gender with a *p* = 0.35. Sensitivity analyses showed that omitting individual studies by Lind GE and Zheng S significantly influenced pooled ORs and CIs for association between p14^ARF^ gene methylation and gender, which resulted in an increase in the pooled ORs from 0.71(95% CI: 0.53–0.95) to 0.75(95% CI: 0.55–1.02) and 0.76(95% CI: 0.56–1.04), respectively (Fig 4A). By omitting Zheng S’s study regarding to the relationship between age and p14^ARF^ methylation, we found that the combined ORs decreased from 3.38(95% CI: 0.76–6.0) to 2.46(-0.73–5.64) (S1E Fig). Similarly, a decrease from 0.72(95% CI: 0.45–1.14) to 0.57(95% CI: 0.34–0.96) was shown when Iacopetta B’s study regarding to the relationship between TP53 mutation status and p14^ARF^ methylation was omitted (Fig 4G).

## Discussion

In our study, we explored whether p14^ARF^ gene methylation was associated with some clinicopathological features of CRC patients through 18 included studies. Results revealed that p14^ARF^ gene methylation was related with the gender of CRC patients (Fig 2A). Although no significant heterogeneity existed between all studies (*I*^2^ = 25.5%, *p* = 0.209), the estimate was not robust in the further leave-one-out analysis. The omitted Lind GE and Zheng S’s studies significantly contributed to this difference in male and female. So, it was hard to conclude that p14^ARF^ methylation is associated with gender in CRC patients. It’s so controversial that more studies are needed to further explore it. Meanwhile, proximal CRC tumors were also found to have a higher possibility of p14^ARF^ gene methylation than distal ones (Fig 2D). These findings that p14^ARF^ gene methylation is more likely to be exhibited in proximal CRC are in line with early studies [14, 25, 27]. This epigenetic alteration pattern may supplement the methylator phenotype for CRC, even supporting the hypothesis that distinct anatomical subtype of CRC exist [34–36]. Although some previous studies hypothesized that cancers of proximal and distal colon were different tumors because they differed in genetic changes, embryologic origin, and biologic identity [37, 38], when it comes to the question why p14^ARF^ tends to be methylated within proximal CRC, deep researches focusing on the mechanism are rare. More related studies are needed to bring it to light.

For lack of unified style of variable, we explored the relationship between age and p14^ARF^ methylation in CRC patients in three separate analyses. Although we observed that p14^ARF^ methylation was more likely inhibited in older CRC patients in one meta-analysis, we couldn’t draw this conclusion easily in the sensitivity analysis. Zheng S’s study significantly contributed to this difference, while all other studies didn’t. When it was removed, difference couldn’t be observed any more. It is well established that age is one of the risk factors for colorectal carcinogenesis. But, is p14^ARF^ more likely to be methylated in older CRC patients? It is hard to say. Maybe we will do more researches about this problem.

Additionally, p14^ARF^ gene methylation was more likely to be present in tumors exhibiting microsatellite instability than those did not, in accordance with previous studies [29, 30, 39]. Most of CRCs probably harbor a defect in the DNA mismatch repair (MMR) system [40, 41], suggesting a link between MMR deficiency and aberrant methylation in CRC. The DNA methyltransferase is known to bind more efficiently to DNA substrates containing mismatched bases than to normal DNA [42, 43], which raises the possibility that it preferentially methylates at these mismatched sites. Another possible mechanism is that expansion of microsatellite may change the local chromatin structure so that the area becomes susceptible to hypermethylation [44]. On the other hand, previous studies have demonstrated that TP53 gene was strong related with MSI [45–47]. However, this relationship was not determined and mechanisms were also not clear. Whether p14^ARF^ methylation influence the status of MSI of CRC patients through its function on p53 expression remains to be figured out.

We also investigated the associations between p14^ARF^ gene methylation and TNM and Dukes stage of CRC. Although study by Dominguez G and colleagues showed that early-stage tumors had a higher p14^ARF^ gene methylation than late-stage ones [11], we did not obtain any similar phenomenon in this study. Furthermore, we evaluated whether p14^ARF^ gene methylation was related with tumor differentiation, and the negative result was consist with most studies [9, 14, 25]. Although p14^ARF^ gene methylation has ever been showed to be preferentially exhibited in CRC possessing the wild type TP53 [32, 39], no such significant association was found in our study when omitting Iacopetta B’s study in the sensitivity analysis. More detailed researches should be carried out to confirm these uncertainties.

All funnel plots did not show any obvious asymmetry (Fig 3). Besides, Peters test was used to confirm the symmetry of funnel plot for eligible studies concerning gender with a *p* = 0.35, which further showed the absence of publication bias. Other funnel plots were not statistically investigated because when there were fewer studies the power of the test was too low to distinguish chance from real asymmetry [48].

To carry out this meta-analysis more scientifically, a comprehensive search method and well defined selection criteria were applied to obtain the eligible studies. Considering the influence of heterogeneity between studies, the Cochran’s Q-statistic and *I*^*2*^ test were used to choose a fixed or random effects model. In order to reduce the bias, publication bias was also estimated by funnel plots and sensitivity analysis was also performed to evaluate the influence of each single study on the overall estimate.

However, limitations should also be noted in this meta-analysis. Firstly, the possibility of information and selection biases could not be completely avoided because all of the included studies were retrospective. Secondly, we failed to obtain the detailed information of methylation locus for lack of data, which hindered us from subgroup analysis for more accurate estimates. Lastly, some included studies did not well predefine the inclusion criteria for patients, which might have influenced our results.

All in all, our meta-analysis indicates that p14^ARF^ gene methylation may be significantly associated with tumor location and MSI status of CRC and may supplement the methylator phenotype of CRC patients.

## Supporting Information

S1 PRISMA Checklist PRISMA 2009 Checklist. (DOC)

S1 Fig The relationship between age and p14^ARF^ gene methylation in CRC patients. (A) (B) (C) Forest plots in three meta-analyses; (D) Funnel plots; (E) Sensitivity analysis.
(TIF)
