# Supplementary figures and images for: Relationships between p14ARF Gene Methylation and Clinicopathological Features of Colorectal Cancer: A Meta-Analysis
Source: PLoS One. 2016 Mar 21;11(3):e0152050. doi: 10.1371/journal.pone.0152050 (PMC4801177; doi:10.1371/journal.pone.0152050)

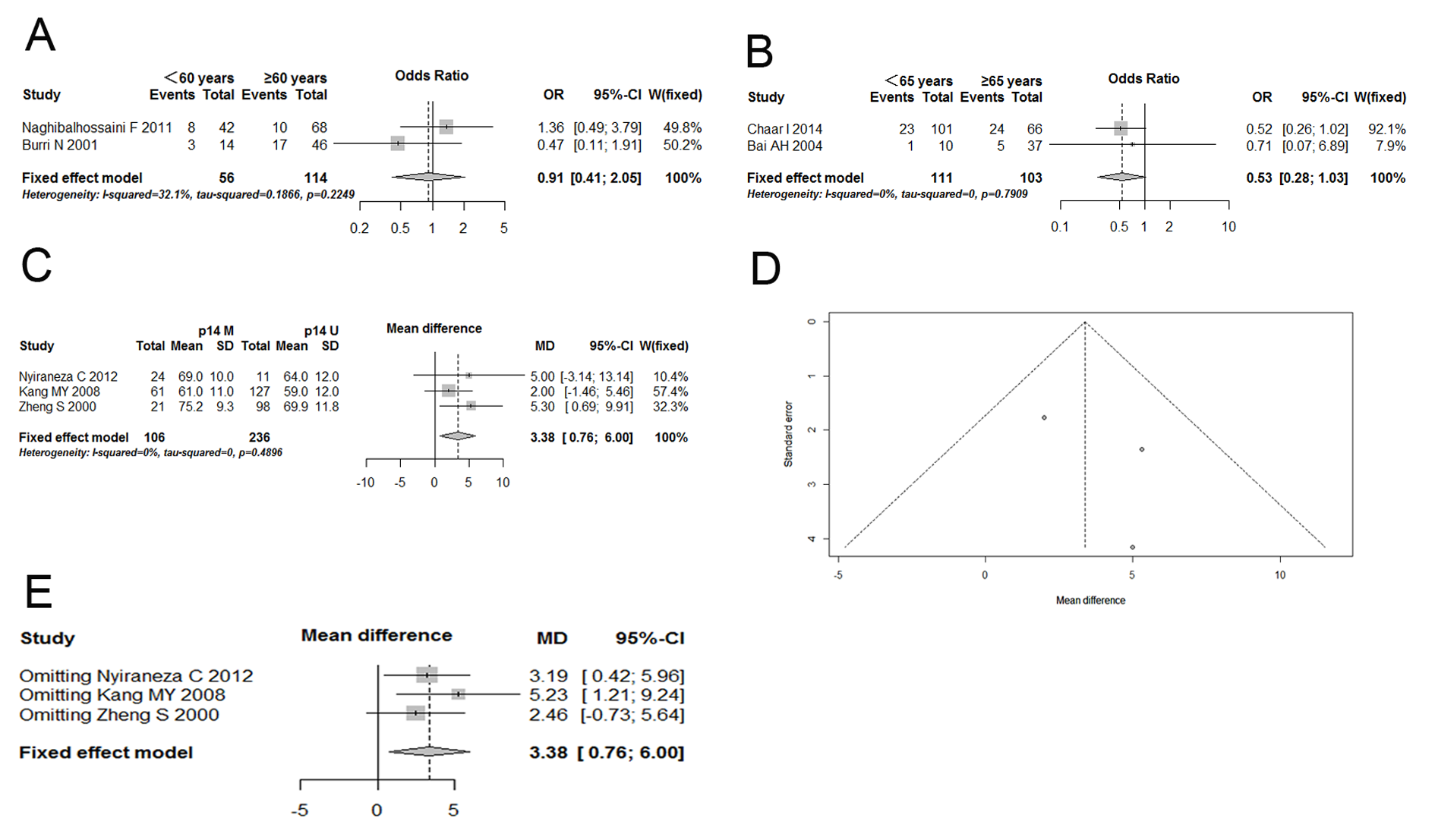

Supplement: S1 Fig — (A) (B) (C) Forest plots in three meta-analyses; (D) Funnel plots; (E) Sensitivity analysis. (TIF) [file pone.0152050.s002.tif]
